# Preoperative acute lung injury and oxygenation impairment occurred in the patients with acute aortic dissection

**DOI:** 10.1186/s12872-022-02579-9

**Published:** 2022-03-27

**Authors:** Xuemin Zhao, Mengjun Bie

**Affiliations:** 1 Department of Cardiology, The First Branch Hospital of The First Affiliated Hospital of Chongqing Medical University, Chongqing, 400016 China; 2 Department of Cardiothoracic Surgery, The First Affiliated Hospital of Chongqing Medical University, Chongqing, 400016 China

**Keywords:** Acute aortic dissection, Acute lung injury, Oxygenation impairment, Inflammatory response

## Abstract

Acute lung injury (ALI) and oxygenation impairment (OI) frequently occur in the patients with acute aortic dissection (AAD), which may necessitate mechanical ventilation and result in adverse outcomes. This paper aims to increase clinicians’ awareness of the severe respiratory complications in the patients with AAD, and provide the overview of the epidemiology, adverse outcomes, pathogenesis, predictive markers and therapeutic modalities of the concurrent conditions. Currently, it is considered that inflammatory response plays a great role in the pathogenesis of ALI and OI in the patients with AAD, but the definite pathogenesis remains unclear. Given the great importance of the prediction of the occurrence of the severe respiratory complication at a very early stage, some inflammatory biomarkers have been investigated to predict the occurrence of ALI and OI in several studies. C-reactive protein was found to have a significant predictive effect for the development of ALI and OI. Early use of beta-blockers and the use of bindarit could prevent the occurrence of OI and ALI. Ulinastatin could also improve oxygenation in the patients with type-A AAD. Prevention and management of ALI and OI in AAD remain a great challenge. The definite pathogenesis should be clearly clarified and further studies should be performed to look for potential effective way to predict and manage the severe respiratory conditions.

## Background

Acute aortic dissection (AAD) is a life-threatening cardiovascular disease associated with high morbidity and mortality. The incidence of aortic dissection is estimated at 4–6 cases per 100,000 person-years [1, 2]. Some fatal complications may potentially occur in the patients with AAD, such as aortic rupture, pericardial tamponade, and multiple organs dysfunction. If untreated, AAD has a mortality rate of 33% within the first 24 h and the mortality rate can rise up to 50% by the first 48 h [3]. Data showed that in-hospital mortality rate was as high as 30% in Stanford type-A AAD and 13% in Stanford type-B AAD [4]. After surgery, the long-term mortality rate ranged from 4 to 48% at 1 year and 9–63% at 5 years for type-B AAD, and similar long-term outcomes could be achieved for type-A AAD [4].

AAD is a systemic disease and some organ damages often occur before operation, such as heart failure, renal and hepatic dysfunction, and neurological deficit. Acute lung injury (ALI) and oxygenation impairment (OI) are critical respiratory conditions, which often occur in the patients with AAD before operation. These preoperative critical complications could result in adverse outcomes [5]. Hence, it is very important to recognize, prevent and manage ALI and OI in the patients with AAD. However, they still remain great challenges in clinical practice. And some doctors are even not aware of the severe respiratory complications.

To our knowledge, there are no definite current guidelines for the pathogenesis, prevention and management of the critical respiratory complications. Limited information has been known on them and therapeutic measurements are usually based on the doctor’s preference. We have comprehensively reviewed the articles focused on this area in the literature with the aim of increasing the knowledge of the critical respiratory complications occurred in the patients with AAD for the doctors and helping them to improve this practice and patient prognosis.

### Clinical characteristics and diagnosis

ALI is the term given to the pathophysiologic presentation of diffuse alveolar injury. It presents as acute hypoxemia with bilateral pulmonary infiltrates on chest imaging that can’t be explained by cardiogenic pulmonary edema [6]. The radiologic hallmarks are the geographic distribution of the patchy ground glass densities, areas of lobular sparing and lower lobe consolidation [7]. The disorder is defined as a syndrome of inflammation and increased pulmonary membrane permeability. Lung compliance is substantially decreased. It can have several etiologies that are systemic, rather than pulmonary and cardiogenic. In 1994, the American-European Consensus Conference Committee established the definition of ALI and acute respiratory distress syndrome (ARDS). The definition included three criteria: (1) the acute onset of diffuse bilateral infiltrates by chest radiography; (2) A PaO_2_/FiO_2_ ratio ≤ 300 for ALI and ≤ 200 for ARDS; (3) Pulmonary artery wedge pressure should be no more than 18, or there is no clinical evidence of left atrial hypertension [8, 9].

For the patients with AAD, ALI often occurs within a few days after the onset of symptoms. To our knowledge, symptoms, signs and radiologic features of the ALI in the patients with AAD have not been descripted in the literature. According to our experience, the symptoms and signs depend on the oxygen levels in blood and the patients’ tolerability for the hypoxemia. The patients may have shortness of breath, tachypnea, and a bluish color in the fingers, lips and toes. If hypoxemia is not severe, some patients may not have any symptoms and signs related to the ALI. The radiological examination is not usually required due to the critical nature of AAD and the results of the laboratory tests are non-specific. ALI is usually diagnosed according to the PaO_2_/FiO_2_ ratio. In the related articles published in the literature, ALI was defined as PaO_2_/FiO_2_ ≤ 300 and OI was defined as PaO_2_/FiO_2_ ≤ 200.

## Epidemiology

There are many reports which estimated the incidence of ALI and OI occurred in the patients with AAD. However, the true incidence is unknown due to the limited sample in each single institution. There are 2 reports on the incidence of ALI complicated by Stanford type-A AAD in the literature, which was 53.8% [10] and 46.5% [11] respectively. And there are 6 articles on the incidence of OI complicated by AAD since the year 2000, which was reported to be 32.2–51% [12–17]. Among the patients with OI, about 31.6–48.4% patients were reported to necessitate mechanical ventilation [14, 15, 17, 18].

## Adverse outcomes

The patients with ALI and OI frequently necessitate mechanical ventilation [17], which may cause ventilator-associated pneumonia and ventilator-associated lung injury [18]. Preoperative OI may result in surgery delay, leading to the increasing risk for higher morbidity and mortality [19]. And some patients may refuse surgery because of the higher perioperative risks estimated. Severe hypoxemia can also occur during surgery even if with the support of mechanical ventilation, increasing the difficulty to maintain satisfactory saturation of blood oxygen for anesthetists. A recent study indicated that patients with ALI had a significant higher morbidity, and the median intubation time, length of stay in intensive critical unit, and length of hospital stay were significantly longer [11]. Gao et al. reported that OI could result in more intraoperative blood loss, longer mechanical ventilation time, and longer stay in intensive critical unit and hospital [13]. Wang et al. reported that preoperative OI is an independent predictor of postoperative hypoxemia (49.5%) in Stanford-A aortic dissection [20]. Hence, it is very important to prevent and manage ALI and OI to decrease the possibility of composite morbidities.

## Pathogenesis

The definite pathogenesis of ALI and OI complicated by AAD remains unclear. However, it is currently considered that systemic inflammatory reactions play great roles in the pathogenesis of ALI and the development of OI. After the aortic injuries, the long-term chronic inflammation in the aortic wall rapidly develops to acute inflammation [15, 18]. A large amount of macrophages infiltration was revealed in the aortic samples from the patients with aortic dissection [21]. And a large number of cytokines including IL-6 and IL-17 have also been found infiltrating the aorta in the patients with aortic dissection [22]. The protective local inflammation may turn to be excessive systemic inflammatory reactions and result in untoward consequences including the organs damage. Additionally, impaired multiple organ perfusion due to the dissection of the entire aorta can also aggravate systemic inflammatory response [23]. Since the pulmonary vascular bed is an important reservoir of neutrophils, the lungs tend to be a major site of tissue damage by AAD [19]. The clinical characteristics of refractory hypoxemia attributes to alveolar edema in inflammatory states when fluid can leak across alveolar-capillary barrier due to the increased permeability [24]. It was suggested that the rapid inflammatory reactions other than the chronic inflammation predominantly contributed to the development of OI [17].

**Fig. 1:**
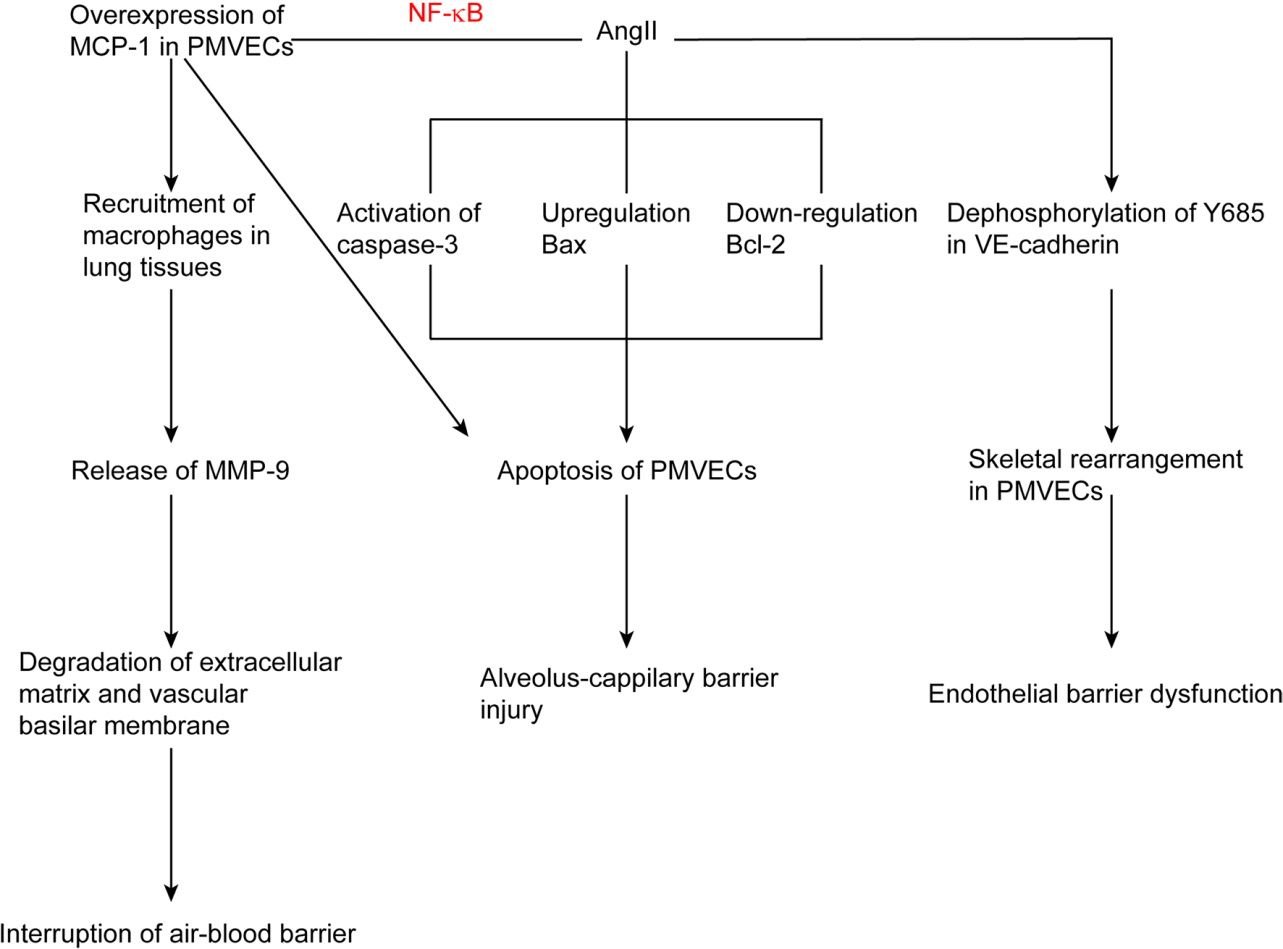
There are three major possible mechanisms of ALI complicated by AAD. (1) AngII could trigger overexpression of MCP-1 in PMVECs by activating NF-κB signaling pathway. MCP-1 plays a great role in the recruitment of macrophages in lung tissues, and MMP-9 derived from macrophages could induce the degradation of extracellular matrix and vascular basilar membrane; (2) AngII could induce the apoptosis of PMVECs through activating the caspase-3, up-regulating the expression of Bax and down-regulating the expression of Bcl-2; (3) AngII could trigger endothelial barrier injury, which may be related to the dephosphorylation of Y685-VE-cadherin and the endothelial skeletal rearrangement. *AngII* angiotensin II, *MCP-1*, monocyte chemoattractant protein-1, *PMVECs* pulmonary microvascular endothelial cells, *MMP-9*, matrix metalloproteinase 9

A few studies have been conducted on the mechanisms of the development of ALI and OI complicated by AAD (Fig. 1). It has been reported that ALI complicated by AAD was highly associated with the macrophages infiltrating the pulmonary interstitial tissues and releasing matrix metalloproteinases 9 [25]. Wu et al. demonstrated that angiotensin II was closely related to the lung injury at the early stage of AAD through mediating the release of matrix metalloproteinases 9 in the macrophages in the lung tissues [26]. Angiotensin II was indicated to induce pulmonary injury by triggering endothelial barrier injury, and such process may be related to the dephosphorylation of Y-685-VE-cadherin and endothelial skeletal rearrangement [27]. Angiotensin II also could induce the production of monocyte chemoattractant-1 and cellular apoptosis in human pulmonary microvascular endothelial cells, which was closely related to the pathogenesis of ALI in AAD [28].

In addition, Gao and his colleagues suggested that coagulation and fibrinolysis appeared to play critical roles in the pathogenesis of OI development [13]. Another study indicated that oxidative stress might be involved in the process of ALI in obese patients with aortic dissection, because obese patients seemed to have high risks for the development of ALI [29].

## Predictive markers

ALI and OI can result in some adverse outcomes, so identification prior to the occurrence of them is very important. The patients with high risks for developing ALI and OI can receive surgical treatment and some other appropriate therapies at the very early stage after the symptom onset. It can help patients gain better prognosis. Hence, it is very necessary to evaluate the predictive value of some biomarkers in the development of ALI and OI complicated by AAD.

A few reports showed the independent association between the patients’ clinical characteristics and OI or ALI development complicated by AAD. Kazunori Tomita and his colleagues demonstrated that non-slender frame, temperature on admission ≥ 36.5 °C and lower oxygenation index on admission were reliable for predicting the occurrence OI in type-B AAD [16]. Yusuke Kashiwagi reported that OI in type-B AAD was correlated with younger age [18]. In OI group, the peak body temperature was also found to be significantly elevated [15, 17]. A recent study indicated that older patients, obese patients and preoperative diastolic blood pressure < 44 mmHg were independent factor related to the occurrence of preoperative ALI in Stanford type-A AAD [10]. It was also suggested that systolic blood pressure and positive smoking history were associated with the occurrence of preoperative ALI in Stanford type-A AAD [11]. However, some other studies did not find any relationship between patients’ demography and OI development [11, 12, 14, 30]. Generally speaking, the solid consensus has not yet been reached on the association between the patients’ characteristics and the development of OI and ALI. Further studies with large samples should be established to gain more reliable conclusions.

Given the vital roles of the inflammatory reactions in the occurrence of ALI and OI in the patients with AAD, some inflammatory biomarkers were investigated to predict the ALI and OI development in AAD. A few studies indicated that the peak levels of C-reactive protein after admission was an independent predictor of OI development [12, 15, 18, 30]. Our previous study also suggested that serum C-reactive protein and albumin measured on admission were independent predictors of OI development in the hypertensive patients with AAD [17]. In the patients with OI, white blood cell levels could be elevated [11, 15]. It has been reported that patients with high mean platelet volume/platelet count ratio had a high risk of preoperative ALI [31]. Some interleukins were also investigated. In 1999, a study indicated that IL-8 might be associated with OI development [32]. A recent study reported that IL-6 and prostaglandin I_2_/thromboxane B_2_ ratio were independent factors related to the occurrence of preoperative ALI in Stanford type-A AAD [11]. Duan et al. also reported that IL-6 could be an independent predictive factor for OI development in Stanford type-A AAD [30].

Based on the important roles of coagulation and fibrinolysis in OI development, a recent study demonstrated that preoperative plasminogen activator inhibitor-1 in bronchoalveolar lavage fluid and tissue factor in both serum and bronchoalveolar lavage fluid were significant factors related to the development for OI in patients with Stanford type-A AAD [13]. Recent studies showed that D-dimer and fibrinogen and fibrin degradation products (FDP) were not associated with the occurrence of ALI and OI in the patients with AAD [17, 18]. AAD% (the percentage of the volume of false lumen to that of aorta in the descending aorta) could possibly show the magnitude of the systemic inflammatory reactions to the aortic injury, and it was demonstrated as the independent predictor of OI in distal type AAD by Manabu Kurabayashi and his colleagues [14]. It provided a non-invasive and easy-to-use alternative to evaluate the possibility of developing ALI and OI at the early stage after admission. Of course, ideal biomarkers should be further introduced to predict the critical respiratory complications with good sensitivity and specificity.

## Therapeutic modalities

Surgery is the mainstay treatment for AAD, but surgical strategy for the patients complicated with ALI and OI has not been established. The operation timing usually accords to the doctors’ preference and no evidence has been reported to determine the appropriate operation timing. Concerning about the adverse outcomes of the respiratory complications on the surgery, doctors sometimes may choose conservative therapy until the state of ALI and OI has been ameliorated. This may lead to the occurrence of some potential fatal complications due to the surgery delay. According to our practical experience, we suggest that surgery for the patients with ALI and OI should be performed at the very early stage after occurrence of them. We guess that it is mainly because surgical closure of the intimal tears may diminish the inflammatory reactions surrounded by the aortic injuries and it may further ameliorate systemic inflammatory reactions. The patients with high risks for developing ALI and OI should also been closely monitored and timely surgical treatment may be helpful to improve prognosis. And the development of ALI and OI should not be the contraindication of the surgery. As we know, it has not been reported about the association between the severe respiratory state and the perioperative survival rate. Detailed data should be reported to help us determine appropriate therapeutic modalities.

Some agents have been investigated to attenuate ALI and OI. Yusuke Jo and his colleagues indicated that use of beta-blockers within 24 h of the onset of AAD could prevent OI [33]. It has been reported that patients with type-A AAD who received ulinastatin at a total dose of 30,000 units prior to surgery could have better oxygenation compared with the patients not receiving ulinastatin [30]. Bindarit, an indazolic derivative, was found to have a significant effect to attenuate the incidence of AAD complicated ALI and inhibit the accumulation of macrophages in lung tissues in rat model, which might be associated with downregulation of the classical nuclear factor kappa-B pathway [34]. Some other studies also provided new insight to look for new potential therapeutic modalities for the concurrent conditions. IL-22 might attenuate ALI induced by angiotensin II because IL-22 contributed to the inhibition of pulmonary microvascular endothelial cells apoptosis mediated by angiotensin II through activating the JAK2/STAT3 signaling pathway [35, 36]. Given oxidative stress and inflammatory response may be involved in the process of ALI of aortic dissection caused by obesity, it may provide new ideas for the treatment of ALI after aortic dissection [29]. It was reported that pretreatment with pravastatin could protect against ischemia-repfusion induced lung injury in an experimental animal model with AAD [37], so potential protective effect of pravastatin for the patients with AAD complicated with OI and ALI may be worthy of investigating in future. Corticosteroids have a wide spectrum of potentially desired actions including anti-inflammatory, antioxidant, pulmonary vasodilator and anti-edematous effects. So we hypothesize that short-term and low-dose administration of corticosteroids should have protective role in the pathogenesis of ALI. And we will investigate the role of corticosteroids in the prevention and therapeutic management of ALI in the patients of AAD in further study.

## Conclusions

ALI and OI are adverse complications frequently occurred in the patients with AAD. Currently the awareness of the severe respiratory conditions is not enough and the information in the literature is relatively limited. It is very difficult to establish guidelines for epidemiology, pathogenesis, prevention, treatment, and prognosis. The management of ALI and OI seems to be blind in clinical practice and it often accords to the doctors’ own preference. Hence, further study should be performed to clarify the pathogenesis of ALI and OI complicated in AAD, and some potential effective ways need to be explored to prevent and manage the ALI and OI.

## Data Availability

The datasets used and/or analyzed in this present study are available from the corresponding author on reasonable request.

## Abbreviations

ALI: Acute lung injury
OI: Oxygenation impairment
AAD: Acute aortic dissection
